# Rapid and powerful detection of subtle allelic imbalance from exome sequencing data with *hapLOHseq*

**DOI:** 10.1093/bioinformatics/btw340

**Published:** 2016-06-10

**Authors:** F. Anthony San Lucas, Smruthy Sivakumar, Selina Vattathil, Jerry Fowler, Eduardo Vilar, Paul Scheet

**Affiliations:** ^1^Department of Translational Molecular Pathology; ^2^The Graduate School of Biomedical Sciences, The University of Texas Health Science Center at Houston, Houston, TX, USA; ^3^Department of Epidemiology,; ^4^Department of Gastrointestinal Medical Oncology; ^5^Department of Clinical Cancer Prevention, The University of Texas MD Anderson Cancer Center, Houston, TX, USA

## Abstract

**Motivation:** The detection of subtle genomic allelic imbalance events has many potential applications. For example, identifying cancer-associated allelic imbalanced regions in low tumor-cellularity samples or in low-proportion tumor subclones can be used for early cancer detection, prognostic assessment and therapeutic selection in cancer patients. We developed *hapLOHseq* for the detection of subtle allelic imbalance events from next-generation sequencing data.

**Results:** Our method identified events of 10 megabases or greater occurring in as little as 16% of the sample in exome sequencing data (at 80×) and 4% in whole genome sequencing data (at 30×), far exceeding the capabilities of existing software. We also found *hapLOHseq* to be superior at detecting large chromosomal changes across a series of pancreatic samples from TCGA.

**Availability and Implementation:** *hapLOHseq* is available at scheet.org/software, distributed under an open source MIT license.

**Contact:** pscheet@alum.wustl.edu

**Supplementary information:** Supplementary data are available at *Bioinformatics* online.

## 1 Introduction

A critical mechanism by which cancer cells operate is through activation of oncogenes or inactivation of tumor suppressor genes. This may happen via acquired chromosomal alterations, such as amplification, deletion or copy-neutral loss-of-heterozygosity (cn-LOH), that result in allelic imbalance (AI). Because AI provides insights into the progression towards malignancy and metastasis, AI detection can be applied to help in cancer prognosis and therapeutic decision-making (Diep *et al.*, 2006).

In the last decade, array comparative genomic hybridization (aCGH) and single-nucleotide polymorphism (SNP) array based approaches have become popular technologies for AI detection (Carter, 2007). More recently, the advent of next-generation sequencing (NGS) technologies for studies of cancer genomic variation (Zhao *et al.*, 2013) has brought the potential for finer resolution (detection and boundary refinement) due to a denser marker set and arbitrarily high coverage. However, costs limit depth of coverage through whole-genome sequencing (WGS) and therefore whole-exome sequencing (WES) is often preferred for genomic studies. WES presents a particular challenge for inferring AI, since coverage is variable across regions and the target is limited to ∼3% of the genome.

Several tools model expected coverage based on summaries from a WES reference panel to account for technical variability in coverage and then infer copy number changes or AI. However, these approaches fail to detect *subtle* chromosomal AI events in NGS data—i.e. aberrations such as amplifications, deletions and cn-LOH occurring in a small fraction (< 30% for WES, <10% for WGS) of the DNA in a heterogeneous sample, scenarios highly relevant to comprehensive tumor profiling and diagnostics. For example, the median tumor cellularity for TCGA pancreatic adenocarcinoma (PDAC) samples is 53% (*n* = 186). Of these, 57 samples (31%) have tumor fractions of 30% or less, which is below AI detection levels of currently available tools (Supplementary Fig. S1). This gap is therefore significant, especially since tissue samples are often limited and additional surveys with complementary technologies such as aCGH may not be possible.

## 2 Methods

*hapLOHseq* is an application for detecting AI events in NGS data, motivated by the logic underlying a method for AI detection in SNP array data (Vattathil and Scheet, 2013). Inputs to *hapLOHseq* consist of a variant call format (VCF) file with an allelic depth (AD) field containing the read depths of the reference and alternate alleles, generated from either WGS or WES, and a set of haplotype estimates. The output from *hapLOHseq* is a list of putative AI regions of the genome built from a detailed report of probabilities for each heterozygous genotype residing in a region of AI.

When assessing high-purity tumor samples for which paired normal samples are available for inferring germline genotypes, one can directly compare genotypes of the tumor and normal samples and clearly characterize copy number changes. However, when the sample contains a high proportion of normal cells (low tumor cellularity), the genotypes called from the tumor sample will reflect the germline rather than the tumor. To extract maximal information from the data, *hapLOHseq* leverages a lower-level data source: the allele-specific read counts. *hapLOHseq* achieves its power by capturing signals among multiple sites jointly (haplotype level) rather than relying on imbalances observed marginally at heterozygous sites. First, a user statistically estimates germline haplotypes from variant sites called in a paired normal sample, or the tumor sample itself when cellularity is low. (For convenience, the *hapLOHseq* download contains a companion phasing utility, allowing direct application to a single VCF.) The AI detection method then: (i) assesses similarity between the observed reference allele frequencies (RAFs) from the NGS data and the haplotypes; and (ii) identifies regions where this similarity achieves statistical significance, indicating haplotype, or *allelic*, imbalance.

Similarity between the alleles in relative abundance and one of the germline haplotypes suggests an imbalance of a segment of an inherited chromosome due to, perhaps, acquired alterations. To assess the correlation, we first determine a putative ‘excess haplotype’ by applying a threshold to the RAFs at each marker independently in a ‘frequency-based phasing algorithm’. By default, the threshold is defined as the median variant allele frequency across the genome (but should be close to 0.5). The alleles with frequencies above the threshold constitute one putative haplotype. Where no imbalance exists, the RAF-based haplotype estimate reflects noise. Otherwise, where AI does exist, the RAF-based haplotypes should bear some resemblance to the statistically estimated haplotypes. This resemblance is quantified with *phase concordance*, a measure of similarity that accommodates errors in the statistical haplotype estimates. A hidden Markov model (HMM) is then applied to assess the spatial aggregation of markers with evidence for haplotype-RAF consistency and to compute a probability of regions of the genome being in AI (Vattathil and Scheet, 2013).

## 3 Results

We obtained the WGS and WES reads for the tumor and paired normal sample of a glioblastoma patient (TCGA-19-2620) from The Cancer Genome Atlas (TCGA) in addition to the SNP-array-based copy number and LOH calls published by the TCGA consortium (Brennan *et al.*, 2013). To assess the sensitivity of *hapLOHseq*, we created *in silico* mixtures of the reads from the tumor and normal BAM files at multiple ratios, mimicking varying tumor purities. The published purity estimate for the original tumor sample was 80%, which we accounted for in our simulations. We created *in silico* mixtures of 4, 8, 12, 16, 20, 28, 40, 56 and 80% tumor.

**Fig. 1.**
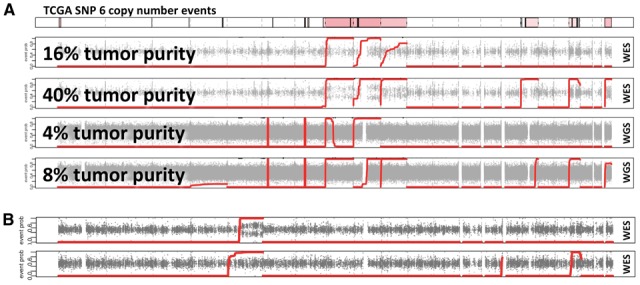
*hapLOHseq* discovers subtle AI in NGS. (**A**) Top panel shows AI events from a TCGA SNP array. Red lines show *hapLOHseq* evidence for AI in WES (16, 40%) and WGS (4, 8%) mixtures. (**B**) WES of two adenomas from an FAP patient; *hapLOHseq* detects AI on chr. 5q

We then applied *hapLOHseq* with default settings to the VCFs created from processing the mixed BAM files, first phasing haplotypes with *MACH* (Li *et al.*, 2010). Results are depicted in Figure 1. In WES data, *hapLOHseq* is able to detect large regions of AI at tumor purities of 16% and virtually all events at 40%. The sensitivity of *hapLOHseq* is greater on WGS data, with all events discovered at 8% purity and some events at a mere 4%. For comparison, we assessed performance of the following exome AI detection tools: *ADTEx*, *FREEC* and *ExomeDepth* (Amarasinghe *et al.*, 2014; Boeva *et al.*, 2012; Plagnol *et al.*, 2012). *hapLOHseq* outperformed these methods (Supplementary Fig. S1). *ADTex*, the best among the other methods, was able to detect events at 28% tumor purity, in-line with its published detectable limit of 30% (Amarasinghe *et al.*, 2014).

We also downloaded and analyzed the exome sequences of 12 tumor-normal pairs of TCGA PDAC samples using *hapLOHseq* and the other tools (Supplementary Fig. S2). *hapLOHseq* consistently outperformed these methods in its ability to efficiently identify chromosomal AI regions of the genome (Supplementary Figs S2 and S3).

Finally, we applied *hapLOHseq* to WES experiments on two adenomas from a patient with familial adenomatous polyposis (FAP), a cancer syndrome resulting from a germline mutation in *APC* (5q), where the acquired second somatic mutation (or ‘hit’; Knudson, 1971) may be an LOH event (Galiatsatos and Foulkes, 2006). Figure 1 shows discovery of AI in both adenomas, where in the second sample there is no visual perturbation of the RAFs.

In summary, *hapLOHseq* is able to detect AI in WES and WGS data at low cell fractions. Further, it does not require a paired normal sample (whereas *ADTEx*, for example, does). Interestingly, we observe sufficiently strong dependency among alleles (linkage disequilibrium) in WES data for our approach to excel. *hapLOHseq* should be useful for the detection and profiling of AI in tumor samples that are either heavily diluted with normal tissue cells or in heterogeneous tumors or premalignant lesions.

## Funding

This work was supported by the Department of Translational Molecular Pathology Fellowship at The University of Texas MD Anderson Cancer Center, NIH grants R03CA176788, U24CA143883, U01GM92666, R01HG005859 and by The University of Texas MD Anderson Cancer Center Core Support Grant.

*Conflict of Interest*: none declared.

## Supplementary Material

Supplementary Data
