# Supplementary material for: Rapid and powerful detection of subtle allelic imbalance from exome sequencing data with hapLOHseq
Source: Bioinformatics. 2016 Jun 10;32(19):3015–7. doi: 10.1093/bioinformatics/btw340 (PMC5039922; doi:10.1093/bioinformatics/btw340)
Supplement: Supplementary Data [file supp_32_19_3015__index.html]

Rapid and powerful detection of subtle allelic imbalance from exome sequencing data with hapLOHseq — Rapid and powerful detection of subtle allelic imbalance from exome sequencing data with hapLOHseq — Supplementary Data 

# Rapid and powerful detection of subtle allelic imbalance from exome sequencing data with *hapLOHseq*

## Supplementary Data

files

- Supplementary Data - docx file
