# Supplementary material for: Rapid and powerful detection of subtle allelic imbalance from exome sequencing data with hapLOHseq
Source: Bioinformatics. 2016 Jun 10;32(19):3015–7. doi: 10.1093/bioinformatics/btw340 (PMC5039922; doi:10.1093/bioinformatics/btw340)
Supplement: Supplementary Data [file supp_btw340_haplohseq_supplemental.docx]

**Rapid and powerful detection of subtle allelic imbalance from exome sequencing data with *hapLOHseq***

**Supplementary Materials**

Samples used in the method evaluations 1

Exome sequencing evaluation method 1

Evaluation results 2

Tools comparisons using the TCGA GBM sample (TCGA-19-2620) tumor dilutions 2

Tools comparisons using the TCGA PDAC exome sequence tumor samples 2

Tools evaluation 3

References 4

## Samples used in the method evaluations

We computationally mixed the exome sequencing reads of the tumor and normal sample for one TCGA Glioblastoma (GBM) sample (TCGA-19-2620) at 5% tumor dilution intervals. We also analyzed TCGA pancreatic adenocarcinoma (PDAC) exome sequencing samples (tumor and normal pairs) of 12 patients. TCGA PDAC samples are characterized by high stromal contamination, as illustrated by the sample metadata at the TCGA Data Portal (<https://tcga-data.nci.nih.gov/tcga>), which showed that the median tumor cellularity for TCGA PDAC samples is 53% (n=186). Of these, 57 samples (31%) have tumor fractions of 30% or less. Aligned reads were downloaded for the tumor and normal exome sequence of each GBM and PDAC at CGHub (<https://cghub.ucsc.edu>).

## Exome sequencing evaluation method

We compared the performance of *hapLOHseq* on exome sequencing data with three tools: *ADTex*, *FREEC* and *ExomeDepth* with respect to their detection of allelic imbalance (AI) events (Amarasinghe, et al., 2014; Boeva, et al., 2012; Plagnol, et al., 2012). We compared the aberrant regions identified in exome sequence data by each tool with AI events identified by the TCGA consortium using SNP arrays. We limited the TCGA calls to events of 1 megabase or greater in size. Chromosomes that were obviously missed by TCGA SNP array calls (i.e., those with obvious visual B-allele frequency band separation across a chromosome) were manually added to the TCGA call set. The resulting TCGA calls are considered the “gold standard” for the performance assessment.

Default settings were used for each tool. Event output files of each tool were converted to BED format and then BEDTools (Quinlan and Hall, 2010) was used to make call comparisons for each tool versus the “gold standard”. An in-house python script was written to summarize the evaluation metrics, including: (1) percentage of events detected by each method (where an event is *detected* if a method calls at least 50% of the true event region as aberrant), (2) percentage of true event bases detected by each method and (3) percentage of called bases that are in true genomic AI regions.

## Evaluation results

### Tools comparisons using the TCGA GBM sample (TCGA-19-2620) tumor dilutions

Here, we illustrate call comparisons of the four tools compared to the TCGA gold standard. Graphs for 4 dilutions that provide insights into the detection levels of the methods are shown. Events identified in computationally diluted tumor samples at increasing tumor fractions show that *hapLOHseq* is able to detect AI events in the samples in as low as a 16% tumor fraction, while *ADTex* (the best performing other tool) was able to detect events starting at a 28% tumor fraction, which is in-line with their published detection level of a 30% tumor fraction (Amarasinghe, et al., 2014).

Figure S1: Events identified in computationally diluted tumor samples at increasing tumor fractions. TCGA-consortium calls are shown in brown, while *hapLOHseq* event probabilities (with a y-axis range of 0 to 1) are shaded in gray. The calls for *ADTex*, *FREEC* and *ExomeDepth* are illustrated in tracks across the genome in blue, red and green respectively.

### Tools comparisons using the TCGA PDAC exome sequence tumor samples

We evaluated the tools on an additional 12 TCGA PDAC exome samples. *hapLOHseq* consistently exhibits the best sensitivity for detecting TCGA-called events, especially in the lower tumor fraction samples (which are on the left of Figure S2). With a few event exceptions (which are likely highly amplified or deleted regions), other tools have difficulty identifying AI events in the lower tumor fraction samples (50% tumor fraction or below) on the left of Figure S2.

Figure S2: Comparison of allelic imbalance calls across PDAC samples ordered by increasing tumor fraction.

### Tools evaluation

We evaluated the AI calls of each method compared to the TCGA-consortium calls for the TCGA GBM tumor (TCGA-19-2620) dilutions (left of Figure S3) and the TCGA PDAC samples (right of Figure S3). Figure S3 A and B show that *hapLOHseq* performs the best in identifying true chromosomal AI regions in both data sets. Further, Figure S3C shows that of the aberrant regions identified by each method, *hapLOHseq* is most efficient, where a higher percentage of its called bases identify true aberrant regions of the genome.

Figure S3: Performance evaluations of each method compared to the TCGA-consortium calls for the TCGA GBM tumor (TCGA-19-2620) dilutions (left) and the TCGA PDAC samples (right). (A) The percentage of the SNP-array based allelic imbalance events identified by TCGA-consortium that was detected by each evaluated method. (B) The percentage of the SNP-array based allelic imbalance *bases* identified by TCGA-consortium that was detected by each evaluated method. (C) The percentage of the aberrant bases called by each method that overlap TCGA-consortium identified events.

## References

Amarasinghe, K.C.*, et al.* Inferring copy number and genotype in tumour exome data. *BMC Genomics* 2014;15:732.

Boeva, V.*, et al.* Control-FREEC: a tool for assessing copy number and allelic content using next-generation sequencing data. *Bioinformatics* 2012;28(3):423-425.

Plagnol, V.*, et al.* A robust model for read count data in exome sequencing experiments and implications for copy number variant calling. *Bioinformatics* 2012;28(21):2747-2754.

Quinlan, A.R. and Hall, I.M. BEDTools: a flexible suite of utilities for comparing genomic features. *Bioinformatics* 2010;26(6):841-842.
